# Large‐scale genetic surveys for main extant population of wild giant panda (*Ailuropoda melanoleuca*) reveals an urgent need of human management

**DOI:** 10.1111/eva.13532

**Published:** 2023-02-05

**Authors:** Wanyu Li, Chuang Zhou, Meiling Cheng, Hongmei Tu, Guannan Wang, Yeming Mao, Yaohua Huang, Minghua Chen, Megan Price, Yang Meng, Bisong Yue, Xiuyue Zhang

**Affiliations:** ^1^ Key Laboratory of Bioresources and Ecoenvironment (Ministry of Education), College of Life Sciences Sichuan University Chengdu China; ^2^ Sichuan Key Laboratory of Conservation Biology on Endangered Wildlife, College of Life Sciences Sichuan University Chengdu China; ^3^ State Forestry and Grassland Administration Key Laboratory of Conservation Biology for Rare Animals of the Giant Panda State Park China Conservation and Research Center for the Giant Panda Dujiangyan China; ^4^ Sichuan Heizhugou National Nature Reserve Administration Ebian China; ^5^ Sichuan Mabian National Nature Reserve Administration Leshan China; ^6^ Sichuan Meigu National Nature Reserve Administration Meigu China

**Keywords:** *Ailuropoda melanoleuca*, conservation, genetic differentiation, genetic diversity, Liangshan Mountains, population size

## Abstract

There are only six isolated living giant panda populations, and a comprehensive understanding of their genetic health status is crucial for the conservation of this vulnerable species. Liangshan Mountains is one of the main distribution areas of living giant pandas and is outside the newly established Giant panda national park. In this study, 971 giant panda fecal samples were collected in the heartland of Liangshan Mountains (Mabian Dafengding Nature Reserve: MB; Meigu Dafengding Nature Reserve: MG; and Heizhugou Nature Reserve: HZG). Microsatellite markers and mitochondrial D‐loop sequences were used to estimate population size and genetic diversity. We identified 92 individuals (MB: 27, MG: 22, HZG: 43) from the three reserves. Our results showed that: (1) genetic diversity of three giant panda populations was moderate; (2) several loci deviated significantly from the Hardy–Weinberg equilibrium and almost all these deviated loci showed significant heterozygote deficiencies and inbreeding; (3) three giant panda populations have substantial genetic differentiation with the most differentiation between MB and the two other populations; and (4) a large amount of giant panda feces outside the three reserves were found, implying the existence of protection gap. These results indicated that under stochastic events, the giant panda populations in Liangshan Mountains are at risk of genetic decline or extinction and urgent need of human management. This study revealed that high attention should be paid to the protection of these giant panda populations outside the Giant panda national park, to ensure their survival in their distribution areas.

## 1 INTRODUCTION

The giant panda (*Ailuropoda melanoleuca*) is a vulnerable species endemic to China. Although China's recent efforts have greatly increased the number and distribution of the wild giant pandas, the species is only distributed in six isolated mountains, namely, Qinling Mountains, Minshan Mountains, Qionglai Mountains, Liangshan Mountains, Daxiangling Mountains and Xiaoxiangling Mountains (State Forestry Administration, 2006). The wild giant pandas are subject to different degrees of habitat fragmentation at each of the six mountains, and is further divided into more than 30 small populations (Loucks et al., 2001; Lu et al., 2001; O'Brien et al., 1994; Qing, 2016). Therefore, the giant panda is still at a great risk of extinction (Sichuan Provincial Forestry Department, 2015), particularly being vulnerable to stochastic processes. And thus a comprehensive understanding the population size and genetic health status of giant pandas in these regions is crucial for the protection decision‐making and conservation of this vulnerable species.

**FIGURE 1:**
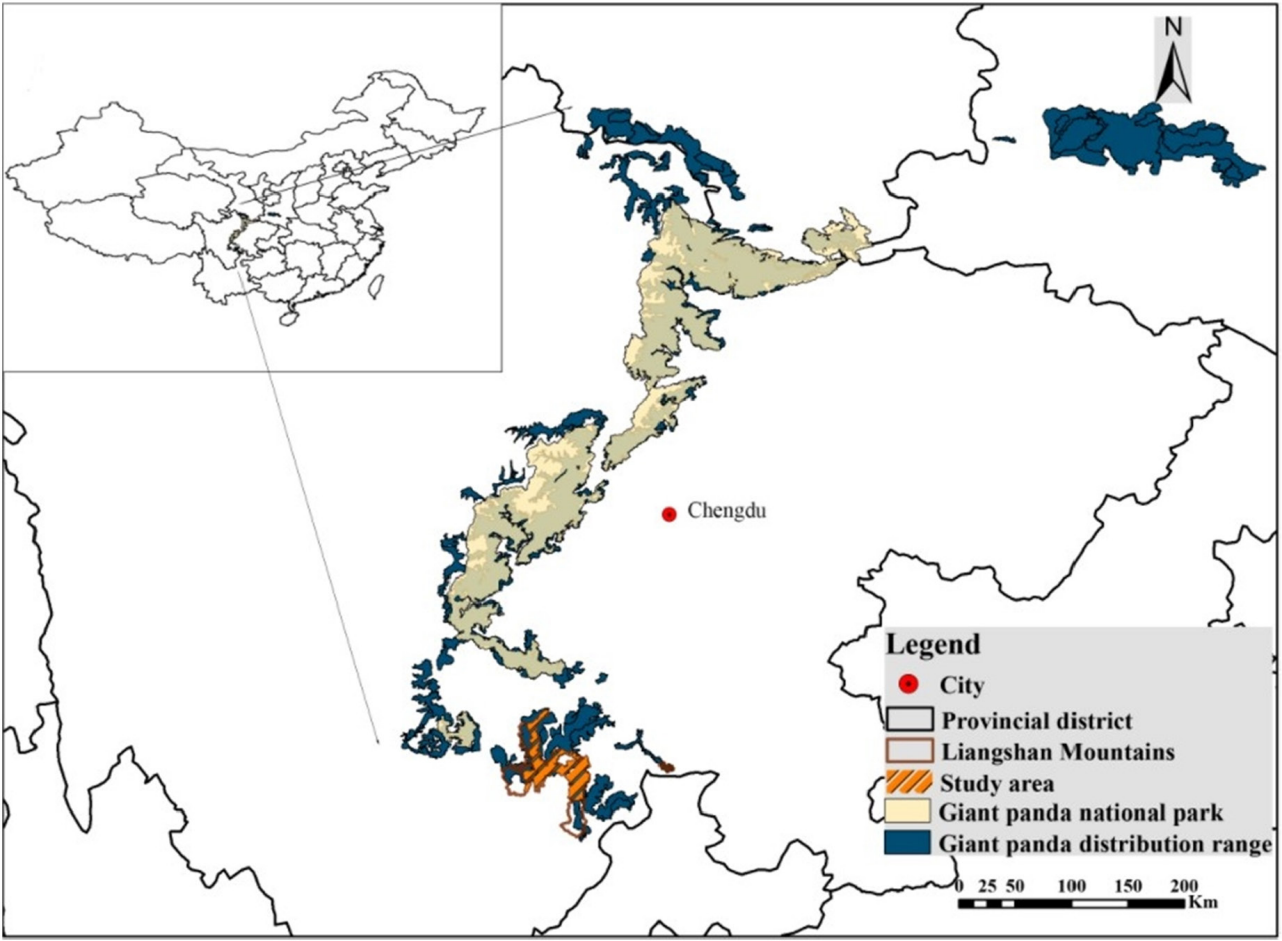
The relative position of the study area in China.

The Liangshan Mountains is the southernmost distribution of giant pandas and is located in the transition zone between the southwest edge of the Sichuan basin and the Qinghai Tibet Plateau. The transition zone is within a global biodiversity hotspot, is highly important for the protection of biodiversity in China and is crucial for giant panda protection (Fan et al., 2010). However, the Liangshan Mountains is outside the newly established Giant panda national park (National Forestry and Grassland Administration (National Park Administration), 2019) (Figure 1). According to the fourth survey report (2011–2014) on giant pandas, there are 124 giant pandas in the Liangshan Mountains and are mainly distributed in Heizhugou, Meigu and Mabian nature reserves (Sichuan Provincial Forestry Department, 2015) (Table 1). These three reserves are located in the heartland of the Liangshan Mountains, and thus are crucial for the protection of giant pandas in the Liangshan Mountains. However, the accurate number, genetic diversity, gene exchange and stable inheritance of panda populations in these key areas remain unclear. Understanding these issues will be vital to the protection of giant pandas in the Liangshan Mountains.

**TABLE 1:** Areas and number of giant pandas of each reserve in Liangshan Mountains (Sichuan Provincial Forestry Department, 2015).

Reserve	Area (hm^2^)	Number
Heizhugou	29,643	29
Meigu	50,655	22
Mabian	30,164	18
Maanshan	27,981	4
Mamize	38,800	3
Laojunshan	3500	3
Shenguozhuang	33,700	2

Microsatellite markers have become an important genetic markers in the field of molecular biology (Selkoe & Toonen, 2010) and have been widely used in population surveys (Creel et al., 2003; Piggott et al., 2006; Wang et al., 2016), genetic diversity assessments (Du et al., 2016; Li et al., 2010; Shen et al., 2010; Vanhala et al., 1998; Zhang et al., 2007), and genetic management of populations (Shan et al., 2014). The combined application of microsatellite markers, mitochondrial markers and noninvasive genetic sampling has contributed greatly to giant pandas conservation in the past 20 years, allowing giant panda population studies without the risk of capture stress, injury or death. Consequently, we used microsatellite markers and mitochondrial markers (D‐loop) to accurately identify population size and assess the genetic traits of giant pandas in Heizhugou, Meigu and Mabian giant panda populations. We aimed to assess the genetic health status of giant pandas and provide reliable data for establishing genetic archives of giant panda populations and developing the genetic management of giant pandas across the Liangshan Mountains. This is the first extensive genetic survey of giant pandas in the Liangshan Mountains.

## 2 MATERIALS AND METHODS

### 2.1 Study area and sample collection

Our study area encompassed Heizhugou, Meigu Dafengding and Mabian Nature Reserves of the Liangshan Mountains (Table 1).

**FIGURE 2:**
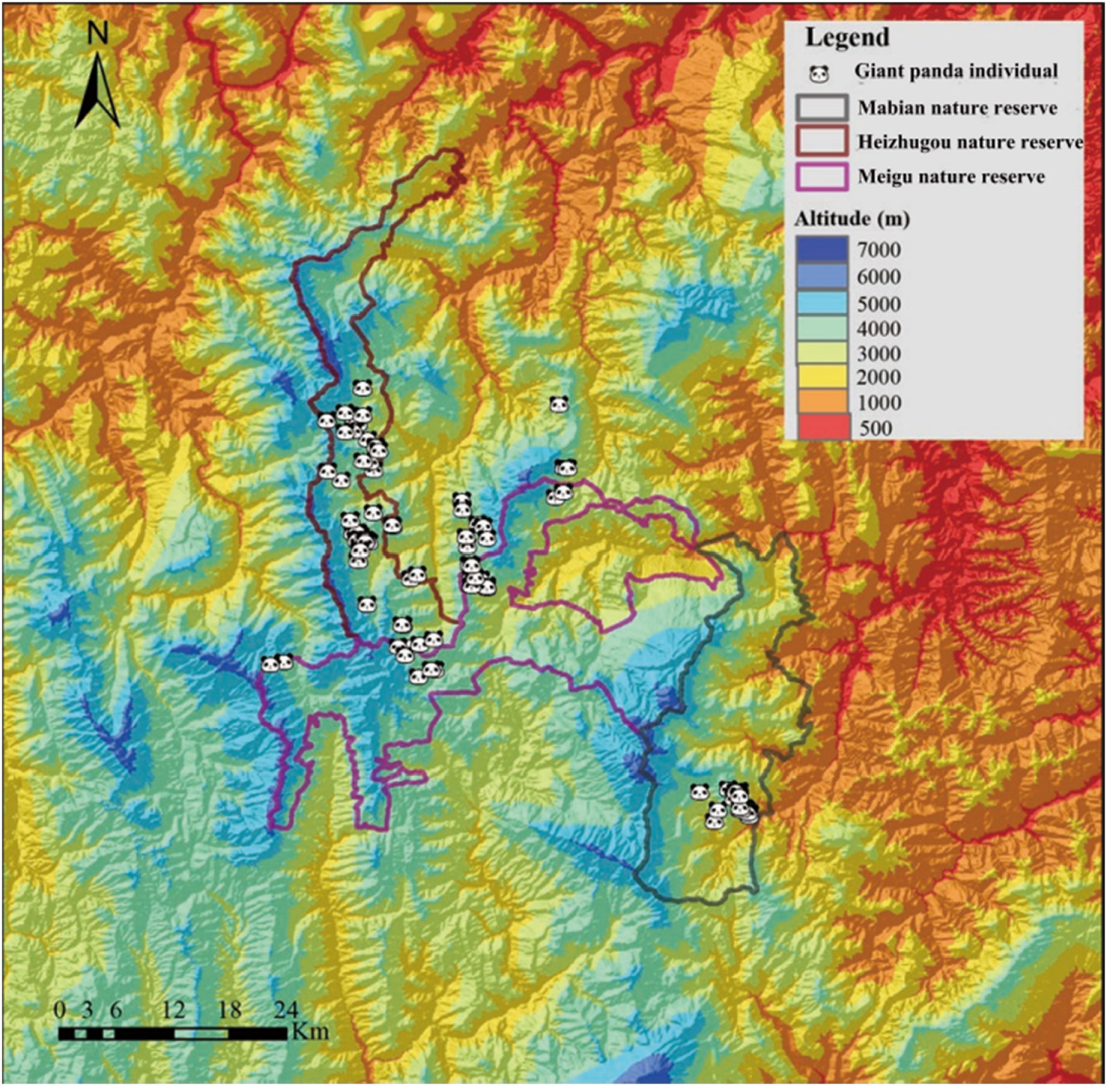
Distribution of identified giant panda individuals within the study area.

Giant pandas fecal samples were collected by ranger staff during their daily monitoring and patrol work in the reserves. The samples were collected from Heizhugou in October 2016 and May 2017, Meigu in October 2017 and May 2018, and Mabian in April and October 2018, respectively. The staff used sterile gloves to collect fresh fecal samples when they detected giant panda activity. Samples were considered fresh based on the color and surface sheen, with dark colored and dull feces being discarded. Each sample was collected in 1–2 copies and stored in a 500 ml sample bottle containing anhydrous ethanol. Spatial coordinates were recorded from the deposition site (e.g., longitude, latitude, elevation) using GPS units and the distribution of samples was mapped as shown in Figure 2, using ArcGIS 10.6 (Price, 2015).

### 2.2 DNA extraction and PCR amplification of mitochondrial D‐loop

Fecal DNA was extracted using the kit (Biobase Upure DNA stool kit) and nucleic acid purifier (Thermo KingFisher). Fecal samples collected in the field were soaked in anhydrous ethanol and frozen at −20°C. DNA extraction was undertaken according to the manufacturer's instructions. The mitochondrial control region was amplified by PCR for those samples where DNA was successfully extracted, using primers from Zhang et al. (2007).

PCR amplifications were performed in a 20 μl reaction volume containing 10 μl 2xTaq PCR Pre Mix (+dye), 1 μl MgCl_2_ (25 mmol/L), 0.8 μl BSA (1 mg/ml), 0.8 μl Ptp primer (15 pmol/L), 0.8 μl BEDH primer (15 pmol/L), 5 μl template DNA (50 ng/μl), and 1.6 μl ddH_2_O. Amplifications were performed using the following PCR procedure: an initial denaturation step for 5 min at 94°C, followed by 40 cycles of 94°C for 50 s, 55°C annealing for 45 s, 72°C elongation for 50 s and a final elongation for 10 min at 72°C. Finally, samples were stored at 4°C.

### 2.3 Selection and amplification of microsatellite markers

Our laboratory has screened giant panda DNA for standardized microsatellite loci and obtained 15 loci that can be effectively applied to giant panda fecal DNA samples (Huang, 2015). We selected seven loci with rich polymorphism, stable amplification and high sensitivity for population analysis, which were GPL8, GPL29, GPL60, gpz20, gpz47, gpy5 and gpy20 (Table 2). Microsatellite amplification and genotyping were performed on fecal samples with successfully mitochondrial sequencing. PCR amplifications were performed in a 20 μl reaction volume comprising 10 μl 2xTaq PCR Pre Mix (+dye), 1 μl MgCl_2_ (25 mmol/L), 0.8 μl BSA (1 mg/ml), 0.8 μlF‐primer (15 pmol/L), 0.8 μl R‐primer (15 pmol/L), 5 μl template DNA (50 ng/μl), and 1.6 μl ddH_2_O. Amplifications were performed using the following PCR procedure: an initial denaturation step for 5 min at 94°C, followed by 40 cycles of 94°C for 50 s, annealing temperature (Table 2) for 45 s, 72°C elongation for 30 s, and a final elongation for 10 min at 72°C. Finally, samples was stored at 4°C. After the PCR amplification, 5 μl of PCR products from each sample was applied to agarose gel electrophoresis with a concentration of 1.5% to detect whether each sample was successfully amplified.

**TABLE 2:** Information on the seven microsatellite loci used in this research.

Locus	Repeat motif	Primer sequence (5′–3′)	Fluorescent tags	Annealing temperature (°C)
GPL60	(TCTT)12	F: TGCCGGAAAGTTCTAAGCAT R: TTTCTCTCCCTCTCCCCTTC	HEX	63
GPL8	(ATCC)11	F: TGGTTTTGCAAGGATGACAG R: TTGTGACAAGCAAGCTCCAC	HEX	63
GPL29	(ATCC)19	F: TCCAAGGCTTCAAACAAGGT R: CACCACAGGTGCCAATTATG	HEX	60
gpz20	(AAAG)10	F: CCCTCTCGTTGTGTCTCTCTG R: CACCTGGTAAATGGCACCTT	HEX	63
gpz47	(AATG)20	F: GACCTCAGTGTACGCCCAGT R: CTGGACAGGCAGGTAGAAGC	HEX	60
gpy5	(AACT)16	F: CTCGGGAGCTTTGTACCATC R: CAGAGAGCCCAAACCTCAAC	HEX	63
gpy20	(TTTG)16	F: GCAGGCACTCAAGAGGTGTT R: CCTTGTGCTAAACACAGGTGA	HEX	63

Microsatellite genotyping was performed at Chengdu Qingke Zixi Biotechnology Co., Ltd. All sequencing was conducted using ABI3730 DNA Analyzer with Genescan 500LIZ size standard (Applied Biosystems). Allele calling was performed using Gene Mapper v4.0.

### 2.4 Data analysis

To minimize the genotyping error in the final individual identification results, we utilized the software Micro‐Checker (Van‐Oosterhout et al., 2004) to examine the presence of null alleles, large allele dropout or stuttering. Individual identification was analyzed using Microsatellite tools (Park, 2001). PID and PID (sib) were calculated using Gimlet (Valière, 2010). The Cervus v3.0 (Marshall et al., 2010) was used to calculate allele number (A), observe heterozygosity (Ho), expected heterozygosity (He) and polymorphic information content (PIC). Deviations from the Hardy–Weinberg equilibrium (HWE) and linkage disequilibrium (LD) were analyzed using Genepop 3.4 (Raymond & Rousset, 1995). Mitochondrial sequence alignment was performed using MEGA v5.2 (Tamura et al., 2011) and was manually calibrated. DnaSP v5.10 (Librado & Rozas, 2009) was used to calculate haplotype diversity (h), nucleotide diversity (π) and other genetic diversity indices. Popgene 32 was used to calculate the inbreeding coefficient of population (Yeh et al., 2000).

Population genetic structure analysis was undertaken using STRUCTURE (Pritchard et al., 2000). The range of possible clusters (*K*) tested was from 1 to 6, and 10 independent runs were carried out for each. The lengths of Markov Chain Monte Carlo (MCMC) iterations and burn‐in were set at 1,000,000 and 100,000, respectively. The true *K* is selected using the maximal value of the log likelihood [Ln Pr(*X*/*K*)] of the posterior probability of the data for a given *K* (Pritchard et al., 2000). The Fst of giant panda population pair‐wise comparisons from the three reserves was calculated and measured by GenALEx 6.5 (Peakall & Smouse, 2012). The gene flow (Nem) among populations was calculated using Nem = (1‐Fst)/4Fst (Wright, 1990), where Nem is the effective number of migrations per generation among populations.

## 3 RESULTS

### 3.1 DNA extraction and amplification of mitochondrial D‐loop region

DNA was extracted from 971 giant panda fecal samples, with a roughly equal sample number from each of the three nature reserves (HZG: 322, MG: 343, MB: 306). We successfully extracted DNA from 731 fecal samples (HZG: 275, MG: 230, MB: 226; Figure S1 for partial electrophoresis).

When collecting fecal samples in the field, the freshness of samples was estimated based on intactness, color, odor, and the status of the mucosal outer‐layer. We estimated most collected fecal samples to be less than 2 weeks old. In addition, even if the fecal freshness is the same, the integrity of the fecal DNA will be different. Mitochondrial DNA is multiple copies and is easier to be amplified compared with nuclear genomic DNA. In this study, the quality of fecal DNA samples was preliminary evaluated by the method of whether the mitochondrial control region of giant panda fecal DNA was successfully amplified or not. A total of 686 DNA samples were successfully amplified for the mitochondrial control region (MB: 218, MG: 228, HZG: 240; Figure S2 for partial electrophoresis) were used to amplify and genotype for seven microsatellite loci.

### 3.2 Individual identification

**FIGURE 3:**
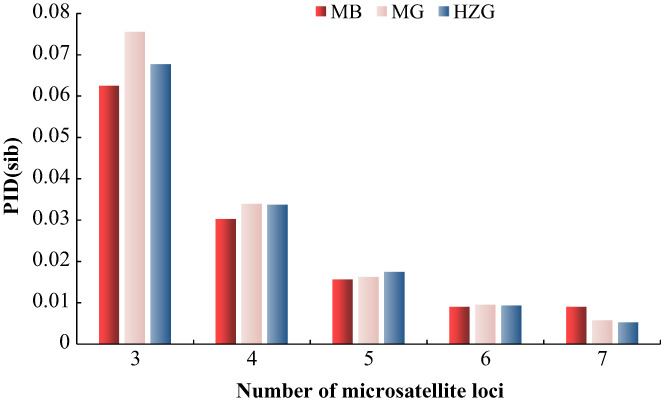
PID (sib) of different number combinations of microsatellite loci.

A total of 406 DNA samples (MB: 81, MG: 60, HZG: 265) successfully completed PCR amplification and genotyping for seven microsatellite loci in this study. Analyses determined that a combination of six loci was most effective for individual identification for the three populations, calculating PID (sib) 0.00911 and 0.00964 (Table 3; Figure 3). According to Waits et al. (2001), a PID less than 0.01 is required to evaluate population size. PID (sib) avoids errors associated with PID and provides a conservative upper estimate of the number of loci required to identify individuals.

**TABLE 3:** Individual recognition simulation results of different combinations of microsatellite.

Loci	3			4			5			6			7		
Reserve	MB	MG	HZG	MB	MG	HZG	MB	MG	HZG	MB	MG	HZG	MB	MG	HZG
PID	4.68 × 10^−4^	9.73 × 10^−4^	1.06 × 10^−3^	8.74 × 10^−5^	1.36 × 10^−4^	2.4 × 10^−4^	1.85 × 10^−5^	2.29 × 10^−5^	5.1 × 10^−5^	6.26 × 10^−6^	7.72 × 10^−6^	1.24 × 10^−5^	6.26 × 10^−6^	2.72 × 10^−6^	0
PIDsib	0.06244	0.07544	0.06765	0.03029	0.03398	0.03377	0.01573	0.01635	0.01757	0.00911	0.00964	0.00945	0.00911	0.00588	0.00537
Number of giant pandas	23	13	39	23	17	43	25	19	43	27	22	43	27	22	43

Abbreviations: HZG, Heizhugou Nature reserve; MB, Mabian Nature reserve; MG, Meigu Nature reserve; PID, Probability of identity; PIDsib, Probability of identity for sibling “Number of giant pandas” is an estimate based on PID.

Individual identification using microsatellites determined that the number of giant pandas in the three reserves was 27 (MB), 22 (MG) and 43 (HZG). Two of the reserves' individual numbers were higher than the fourth survey (18 (MB), 22 (MG) and 29 (HZG)). In addition, two individuals in the HZG were immigrants from MG.

### 3.3 Genetic diversity based on microsatellite markers

Seven microsatellite markers were successfully amplified from the three populations. A total of ninety‐six alleles were detected at seven loci in 92 individuals from the three populations. The number of common alleles in the three populations was 13, but the 13 common alleles in each population were different. We identified five unique alleles in each of Mabian and Meigu populations. Similarly, these two populations had the same number of rare alleles (low frequency alleles) (9). The numbers of unique and rare alleles in Heizhugou population were 15 and 13, respectively. These rare alleles are at risk of being lost due to inbreeding or genetic drift.

The number of alleles at each locus ranged from 1 to 10. The average number of alleles in Heizhugou population was the largest, followed by Meigu population and Mabian population. The average observed heterozygosity (Ho) of the three populations was 0.632 (MB), 0.598 (MG), and 0.466 (HZG), the average expected heterozygosity (He) was 0.577 (MB), 0.502 (MG), and 0.555 (HZG), and the polymorphic information content (PIC) was 0.514 (MB), 0.441 (MG), and 0.508 (HZG), respectively (Table 4). Therefore, the three populations showed a moderate level of genetic diversity. The HWE test results showed that four of the seven microsatellite loci in Mabian population deviated from HWE (*p* < 0.01), while three in Meigu population and two in Heizhugou population (Table 4). A positive mean inbreeding coefficient (Fis) value was found in Heizhugou population (Table 4). High inbreeding coefficient suggests a heterozygote deficiency due to inbreeding. Our results are similar to Guan et al. (2009), who concluded that their observed HWE deviation was due to inbreeding and genetic drift.

**TABLE 4:** Genetic diversity analysis of giant panda population based on microsatellite markers.

	MB							MG							HZG						
	A	N	Ho	He	PIC	Fis	*p*	A	N	Ho	He	PIC	Fis	*p*	A	N	Ho	He	PIC	Fis	*p*
GPL‐8	5	27	0.571	0.579	0.509	0.1072	0.0075	2	22	0.383	0.464	0.354	0.1664	0.1702	3	43	0.535	0.621	0.539	0.1283	0.1672
GPL‐29	5	27	0.821	0.727	0.664	0.1234	0.2614	4	22	0.933	0.67	0.6	−0.1883	0.0006	6	43	0.721	0.788	0.747	0.0740	0.0012
GPL‐60	5	27	0.714	0.764	0.708	−0.2770	0.0066	5	22	1	0.678	0.619	−0.4876	0.0005	5	43	0.465	0.578	0.532	0.1852	0.2432
gpz‐20	8	27	0.786	0.801	0.755	−0.2096	0.0000	7	22	0.717	0.755	0.71	0.0432	0	10	41	0.634	0.792	0.756	0.1894	0.0005
gpz‐47	1	27	0	0	0	—	1	3	22	0.367	0.308	0.268	−0.1348	1	5	43	0.186	0.176	0.167	−0.0717	1
gpy‐5	3	27	0.821	0.645	0.562	−0.4776	0.0019	4	22	0.1	0.128	0.124	0.2140	0.0459	5	43	0.465	0.564	0.486	0.1663	0.2271
gpy‐20	3	27	0.714	0.525	0.4	−0.2054	0.083	3	22	0.683	0.51	0.413	−0.4782	0.0398	4	43	0.256	0.369	0.329	0.2977	0.0479
Average	4.29	27	0.632	0.577	0.514	−0.1565	—	4	22	0.598	0.502	0.441	−0.1236	—	5.4	43	0.466	0.555	0.508	0.1385	—

*Note*: Number of alleles per locus (A), Number of samples (N), observed heterozygosity (Ho), expected heterozygosity (He), polymorphism information content (PIC), Wright's inbreeding coefficient (Fis) and probability of significant deviation from Hardy–Weinberg equilibrium (*p*) are given for each population and locus. Calculations assume that individuals with one microsatellite band are homozygous for the allele.

Abbreviations: HZG, Heizhugou Nature reserve; MB, Mabian Nature reserve; MG, Meigu Nature reserve.

### 3.4 Genetic diversity based on mitochondrial control region sequence

We successfully sequenced the mitochondrial D‐loops from 85 of the 92 individuals from the three reserves, with sequencing peaks shown in Figure S3. The number of mitochondrial D‐loop sequences (n), haplotype (H), variation sites (s), haplotype diversity (h), and nucleotide diversity (π) of the three populations are summarized alongside other wild and captive populations in Table 5. Compared with other populations, the mitochondrial genetic diversity of giant pandas in these three reserves was significantly lower than in wild giant panda populations from Qinling, Minshan and Qionglai Mountains (Yang, 2013). Mitochondrial genetic diversity of three populations was also lower than captive populations from Wolong, Chengdu and Shaanxi, but higher than Daxiangling and Xiaoxiangling populations (Yang, 2013).

**TABLE 5:** Comparative analysis of genetic diversity of giant panda populations based on mitochondrial control region sequences.

Populations	n	H	s	h	π	Reference
Qinling	36	8	6	0.721	0.00335	Yang (2013)
Minshan	44	12	7	0.662	0.00361	
Qionglai	70	20	14	0.744	0.00339	
Liangshan	34	6	3	0.579	0.00216	
Mabian	27	4	4	0.276	0.00094	–
Meigu	20	4	5	0.553	0.00211	–
Heizhugou	38	3	5	0.582	0.00248	–
Xiaoxiangling	32	5	6	0.532	0.0018	Yang (2013)
Daxiangling	21	3	2	0.186	0.00033	
Chengdu	50	6	5	0.604	0.00188	
Wolong	61	16	11	0.632	0.00312	
Shanxi	11	7	7	0.873	0.00394	

Abbreviations: H, Haplotype; h, Haplotype diversity; n, Number of samples; s, Variation site; π, Nucleotide diversity.

### 3.5 Geographic isolation and genetic differentiation

According to the distribution map of giant panda fecal samples (Figure 2), feces collected in Heizhugou and Meigu Nature Reserves were often in close proximity to the border between the two reserves, a few meters from the border, and even quite a few feces were collected at the border line. The mean distances between the main collection sites in each of the three reserves was, respectively, 13 Km (MG‐HZG), 34 Km (MG‐MB), and 45 Km (HZG‐MB). Samples collected in Mabian Nature Reserve were far away from collection sites in Meigu and Heizhugou Nature Reserves.

**FIGURE 4:**
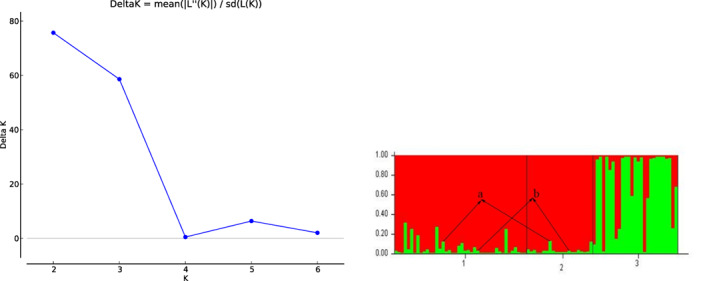
STRUCTURE analysis results of giant panda populations in Heizhugou, Meigu and Mabian Nature Reserves. Delta *K* = mean(|L(*K*)|)/sd(L(*K*)), the corresponding *K* value at the peak of Delta *K* is the optimal *K* value. STRUCTURE output of two genetic clusters identified (*K* = 2), represented by the colors red and green. Each individual is represented by a vertical line, and different color length indicate the probabilities of being assigned to different clusters. (1. Heizhugou; 2. Meigu; 3. Mabian; a and b: two individuals in common).

The software STRUCTURE (Pritchard et al., 2000) was used to analyze the population genetic structure. Our results showed that when *K* = 2, the value of *K* peaked and decreased with increasing values of *K*. As shown in Figure 4, the giant pandas of three reserves were clearly divided into two genetic structural units, Heizhugou and Meigu populations formed a genetic structural unit, while the Mabian population formed a relatively independent genetic structural unit. Thus, the two genetic structural units indicated that the gene exchange between Heizhugou and Meigu populations was more frequent than with the Mabian population.

The analysis of population genetic differentiation (Fst) showed that there was a significant genetic differentiation between the three giant panda populations, with Fst ranging from 0.0756 to 0.1588 (Table 6). The Mabian population had a significantly higher degree of genetic differentiation with Meigu and Heizhuguo population, while there is a moderate degree of differentiation between Heizhuguo and Meigu population. The Nem of the three wild giant panda populations was relatively low, ranging from 1.32431 to 3.05907 (Table 6). Among them, the Fst between Mabian and Meigu was the largest, resulting from the smallest number of effective migrants exchanged per generation (Nem = 1.32431) (Table 6).

**TABLE 6:** F‐statistics (*F*_st_, below the diagonal) and Nem (above the diagonal) of three giant panda populations.

Population	HZG	MG	MB
HZG	–	3.05907	1.62688
MG	0.07555*	–	1.32431
MB	0.13320*	0.15880*	–

* Denotes significant difference (*p* < 0.05).

## 4 DISCUSSION

### 4.1 Population census

Accurate population census is especially complex and important for giant pandas that they are not readily visible and easily counted in their habitat environments. Population census has served as a basis for judging not only the conservation status of pandas but also the effectiveness of measures designed to protect them and their habitat (Wei et al., 2012). Traditionally, an approach using bamboo bite length has been applied. However, the precision of this approach was always known to be low.

In recent years, the development of molecular biology techniques has provided opportunities for more accurate population census. These techniques are mainly carried out at the DNA level, but the biggest obstacle of DNA analysis for the wild panda is the collection of samples (Li et al., 2001). As opposed to both destructive sampling and nondestructive sampling, the researchers realized that noninvasive sampling was ideal for studying the giant panda (Wei et al., 2012). Zhan et al. (2006) proposed a more robust approach to individual identification based on microsatellite amplified from fecal DNA samples of giant pandas, and collected 301 fresh fecal samples in a key panda reserve (Wanglang) and made a molecular census of 66–72 individuals, more than doubling the previous estimate of 27 individuals, suggesting the underestimation of traditional population census.

Similarly, the population censuses of the three reserves in this study are larger than the results of the latest giant panda National Survey (2011–2014). Identification of larger populations in MB and HZG may have two causes, not necessarily mutually exclusive. The first being that different methods were used for our study and the fourth national survey. The fourth national survey predominantly used the “Distance‐Bamboo Stem Fragments Method” adopted in the third national survey (1999–2002) (Shi et al., 2016). The fourth national survey did also employ noninvasive DNA quantity survey technology, but only as an auxiliary survey. Therefore, the fourth national survey may have also underestimated the number of giant pandas. Second, the population's emigration, immigration, births and deaths may give rise to the differences in population number estimation. For example, we detected two individuals in Heizhugou Nature Reserve coming from Meigu Nature Reserve. At the same time, our study excluded two possible sources of error in molecular censuses proposed by Garshelis et al. (2008), namely, lack of geographical closure and genotyping error. We took a few years with the assistance of reserve personnel and extensively collected giant panda feces in three reserves in different seasons. Full coverage of the collection range was achieved both inside and outside the reserves to obtain fecal samples of almost all giant pandas in three reserves. The quality of the feces was assessed before the experiment, the genotyping results were corrected and confirmed, and the correct genotyping data were pooled to build a panda microsatellite database. Therefore, such high‐intensity fecal collection involve almost all giant panda individuals in the reserve, coupled with the sensitive individual identification of microsatellite, the population census in this study should be accurate and credible.

### 4.2 Genetic health assessment of populations

Although genetic diversity is merely one of a number of important considerations in species conservation, the protection of species genetic diversity has always been the core of species protection (Frankham, 2005). The evaluation of the genetic diversity within the protected species can allow conservation practitioners to predict the probability of population extinction or survival when under stress and to provide an theoretical basis for the effective conservation of population. As an extranuclear genetic material, mitochondrial DNA is maternal origin, rapidly evolving, and polymorphic, making it a common molecular marker for species evolution and genetic diversity studies. However, due to maternal inheritance, the lack of recombination, different rate of evolution compared with nuclear DNA, and more sensitive to founder effects and small populations, the results of mitochondrial DNA analysis might be partial and inconsistent with that of nuclear DNA (Barton & Hewitt, 1985; Qin et al., 2017). In this study, to improve the reliability and authenticity of the conclusion, both SSR molecular markers and mitochondrial DNA markers were used to assess the genetic diversity of giant panda population.

The study found that the genetic diversity of Mabian (Ho = 0.6324, He = 0.5773), Meigu (Ho = 0.598, He = 0.502), and Heizhugou (Ho = 0.466, He = 0.555) populations were lower than the diversity of Wolong wild giant panda population (Ho = 0.644, He = 0.684) and Shaanxi captive population (Ho = 0.610, He = 0.593) (Huang, 2015), but higher than the diversity of the wild Qinling Mountains population (Ho = 0.451, He = 0.439) (Ji, 2014). Compared with other threatened species, such as Snow Leopard (*Panthera uncia*) (He = 0.759, Zhou et al., 2015) and forest musk deer (*Moschus berezovskii*) (He = 0.8–0.9, Zou et al., 2005), Liangshan mountains wild giant panda populations had a relatively lower He, but were approximately equivalent to Sichuan sika deer (*Cervus sichuanicus*) (He = 0.562, Ya et al., 2014), and Przewalski's gazelle (*Procapra przewalskii*) (He = 0.552, Ji & Jiang, 2011). Haplotype diversity (h) and nucleotide diversity (π) are two important indicators to measure the level of population genetic variation. We found that mean h and π values from the three reserves were significantly lower than Qionglai, Qinling and Minshan wild giant panda populations, and also lower than Wolong, Chengdu and Shaanxi captive populations, only higher than that of Daxiangling and Xiaoxiangling wild giant panda populations (Table 5). Genetic diversity analysis based on microsatellite markers and mitochondrial control region sequences showed that the genetic diversity level of giant pandas in three Liangshan mountains populations was at a moderate level, and the presences of rare alleles and inbreeding may further reduce their genetic diversity levels. These results show it is necessary to introduce new genetic resource into the three populations or enhance gene exchange between the three populations and/or other populations.

Serious genetic imbalance may lead to the loss of genetic diversity and population decline (Kvist et al., 2015). The Hardy–Weinberg equilibrium is often used as an assessment of genetic balance within a population (Guo & Thompson, 1992). The Hardy–Weinberg equilibrium test results showed that four of the seven microsatellite loci in the Mabian population deviated from the Hardy–Weinberg equilibrium (*p* < 0.01), while three deviated in the Meigu population and two deviated in the Heizhuguo population. Almost all loci that deviated from the Hardy–Weinberg equilibrium showed significant heterozygote deficiencies and significant inbreeding. Inbreeding may be the main cause of deviations from the Hardy–Weinberg equilibrium. Our results showed that the three giant panda populations, especially Mabian, are genetic disequilibrium and there is the risk of further loss of genetic diversity.

Fecal samples were most frequently collected in roughly two geographical clusters. Feces that were frequently found in Mabian reserve were far away from these collection sites of Feces in Meigu and Heizhugou reserves. This geographical clustering was reflected in genetic structural units and differentiation of the three giant panda populations. The giant pandas in three reserves were clearly divided into two genetic structural units. The Meigu and Heizhugou populations formed a genetic structural unit, while the Mabian population was a relatively independent genetic structural unit (Figure 5). Further support for observed clustering was the high genetic differentiation and low gene flow of Mabian population (Fst: 0.13320, 0.15880) with the other two populations. The gene flow in Heizhugou and Meigu, which were close to each other, reached the maximum (3.05907). The genetic clustering also confirms that the geographical clusters were likely indications of higher panda activity and not an effect of sampling method. However, Hu et al. (2010) found that the giant pandas in the Liangshan Mountains lack of genetic differentiation, which is different from our research results. This difference may be caused by different sampling size (156 vs. 406 samples analyzed), DNA sample quality.

The genetic and geographical clustering of the three populations suggests that there is a barrier preventing genetic exchange between the two areas. Feng (2015) found that suitable habitats were fragmented in central and northern Mabian Nature Reserve. Unsuitable habitats might be caused by deforestation, road construction and livestock invasion (Feng, 2015; Zhang et al., 2018; Zhao et al., 2017). Fragmented suitable habitats and unsuitable habitats could influence the habitat selection and migration of giant panda. These unsuitable habitats are mainly concentrated in the western margin and northern sections of the Mabian Nature Reserve (Feng, 2015) and this resulted in giant panda have moved southward. This change might has occurred between the 3rd (1999–2002) and 4th (2011–2014) national panda surveys because the distribution of giant pandas in Mabian moved southward at 4th national panda surveys compared with 3rd surveys (Sichuan Provincial Forestry Department, 2015; State Forestry Administration, 2006). This increased geographically distance and potentially barrier effect between Mabian population and other two populations formed the genetic isolation of Mabian population from other two populations.

Conclusively, the level of genetic diversity of three giant panda populations was moderate, while the genetic diversity of Mabian giant pandas was the lowest. The existence of genetic isolation, a high number of rare alleles, inbreeding and significant deviations from the Hardy–Weinberg equilibrium indicated that these three populations were genetically unstable, and inbreeding may further result in the loss of genetic resources (Wang, 2019).

### 4.3 Genetic management recommendations

Liangshan Mountains is one of the main distribution areas of living giant pandas and belongs to the southernmost distribution of giant pandas. Mabian, Meigu and Heizhugou reserves are located in the heartland of Liangshan Mountains, and are also the core distribution areas of giant pandas in the Liangshan Mountains. The effective protection of the three giant panda populations is crucial for the conservation of all Giant pandas in Liangshan Mountains. The results of our have shown that the three giant panda populations are at risk of decline or extinction given stochastic events, especially the Mabian population. It is therefore urgent to improve each population's genetic status by increasing genetic resources. We recommend two strategies for improving the genetic status of three populations. Firstly, improve genetic diversity of three populations by the introduction of genetically distinct individuals. The China Conservation and Research Center for the Giant Panda and the Chengdu Research Base of the Giant Panda have the largest captive breeding populations of giant pandas in China. These captive populations are genetically stable and distantly related to populations from the Liangshan Mountains (Shan et al., 2014). Therefore, genetic rescue from the two captive breeding populations would increase genetic resources into these core populations of Liangshan Mountains giant panda. However, captive‐bred introductions are difficult and require considerable resources and time (Yang et al., 2018), and therefore it should not be the only strategy for the improvement in genetic health.

Our second recommendation for improving the genetic status of the three populations is to increase connectivity and genetic exchange between the two geographically and genetically distinct panda groups. Although significant genetic differentiation between the two groups exists, no significant difference in behavior and morphology has been found. Similarly, there was no evidence that the Mabian population was subject to different geographical or climatic conditions and thus no unique or local adaptation. Therefore, there should be no genetic, behavioral or morphological impediment to breeding and risk of intraspecific hybridization (Frankham, 2010). The fecal sample distribution (Figure 2) and population genetics demonstrated there was limited genetic exchange between Mabian and two other populations. However, there is no topographical barrier between the two groups, and the limiting factor is likely from unsuitable habitat and habitat fragmentation due to disturbance and lack of bamboo vegetation (Feng, 2015; Zhang et al., 2018; Zhao et al., 2017). Consequently, we recommend that suitable habitat and continuity should be rehabilitated and restored. Recent roads should be reforested or add migration channels for pandas and prevented from new construction. Human activities, especially grazing and bamboo shoot collection, should be controlled and minimized. Existing natural forest (bamboo) should be protected from further damage and the nonbamboo areas should be rehabilitated. As a priority, restoration should focus on creating corridors through the “habitat barrier” to increase panda movement as soon as possible and then expand the area and proportion of suitable habitat. Given that pandas begin moving and they breed, there should be an improvement in the genetic health and population stability of giant panda in Liangshan Mountains.

Although Wei et al. (2020) concluded that China's Panda Protection System and nature reserves can achieve the goals of protecting their habitats and biodiversity, and most giant panda nature reserves have been established based on the distribution of giant pandas, however, the gaps, overlapping designations and disparities in management still exist (Xu et al., 2017, 2019). The reserves in the Liangshan Mountains were established early in China's panda protection efforts and zoning was determined roughly according to predicted panda distributions and human activities. However, many factors have changed over time, and pandas have become more flexible in their habitat choices than previously thought (Hull et al., 2014). For example, space utilization by giant pandas gradually expanded outward between the third and fourth surveys. In addition, we found that a large amount of panda activity occurred outside the reserve (Figure 2), indicating gaps in the coverage of the reserve. Although the Giant panda national park offers an opportunity to promote more effective management and improve the management system by integrating and expanding the existing reserves, however, Liangshan Mountains is not included in the newly established Giant panda national park (National Forestry and Grassland Administration (National Park Administration), 2019). In this case, greater attention should be paid to the protection of the main extant population of wild giant panda. We strongly suggested that the scope of nature reserves in the Liangshan Mountains should be adjusted, by integrating surrounding suitable habitats into the reserve, better protect giant panda habitats, restore degraded habitat, increase gene exchange between populations, and ensure the population stability of giant pandas in Liangshan Mountains.

In conclusion, giant panda populations in Liangshan Mountains had moderate genetic diversity, with a high number of rare alleles, significant heterozygote deficiencies and inbreeding. Three populations clustered into two geographically and genetically distinct groupings, with the Mabian population being separated from the other two by a large tract of unsuitable habitat. The giant panda population in Liangshan Mountains is genetically unstable and at risk of decline or extinction given stochastic events. It is therefore recommended that connectivity between populations be re‐established by improving habitat quality and continuity, and genetic health be enhanced by the introduction of captive‐bred distantly related individuals. These changes could be incorporated into the updated conservation plans for the Liangshan Mountains. Our study revealed that high attention should be paid to the protection of these giant panda populations outside the Giant panda national park, to ensure their survival in their distribution areas, and can serve as a reference for the genetic management of Giant panda populations in other distribution areas and some key conservation species in China and world.

## CONFLICT OF INTEREST

The authors declare no competing interests.

## Supporting information


Figures S1‐S3

## Data Availability

Haplotype sequences were deposited in the GenBank with the accession number OQ108856‐OQ108866.

## References

[eva13532-bib-0001] Barton, N. H. , & Hewitt, G. M. (1985). Analysis of hybrid zones. Annual Review of Ecology and Systematics, 16, 113–148.

[eva13532-bib-0002] Creel, S. , Spong, G. , Sands, J. L. , Rotella, J. , Zeigle, J. , Joe, L. , Murphy, K. M. , & Smith, D. (2003). Population size estimation in Yellowstone wolves with error‐prone noninvasive microsatellite genotypes. Molecular Ecology, 12(7), 2003–2009. 10.1046/j.1365-294X.2003.01868.x 12803649

[eva13532-bib-0003] Du, Y. R. , Zou, X. Y. , Xu, Y. T. , Guo, X. Y. , Li, S. , Zhang, X. Z. , Su, M. Y. , Ma, J. B. , & Guo, S. C. (2016). Microsatellite loci analysis reveals post‐bottleneck recovery of genetic diversity in the Tibetan antelope. Scientific Reports, 6, 35501. 10.1038/srep35501 27739522 PMC5064351

[eva13532-bib-0004] Fan, L. Q. , Dong, L. , Zhang, S. L. , Ran, J. H. , & Yue, B. S. (2010). Landscape pattern of Giant panda habitat in the Liangshan Mountains, Sichuan, China. Chinese Journal of Applied and Environmental Biology, 16(2), 179–184. 10.3724/SP.J.1145.2010.00179

[eva13532-bib-0005] Feng, Z. R. (2015). Assessment on the suitability of giant panda's habitat in Mabian Dafengding nature reserve. Northeast Forestry University.

[eva13532-bib-0006] Frankham, R. (2005). Genetics and extinction. Biological Conservation, 126(2), 131–140. 10.1016/j.biocon.2005.05.002

[eva13532-bib-0007] Frankham, R. (2010). Challenges and opportunities of genetic approaches to biological conservation. Biological Conservation, 143, 1919–1927. 10.1016/j.biocon.2010.05.011

[eva13532-bib-0008] Garshelis, D. L. , Wang, H. , Wang, D. J. , Zhu, X. J. , Li, S. , & McShea, W. J. (2008). Do revised giant panda population estimates aid in their conservation? Ursus, 20, 168–176.

[eva13532-bib-0009] Guan, T. L. , Zeng, B. , Peng, Q. K. , Yue, B. S. , & Zou, F. D. (2009). Microsatellite analysis of the genetic structure of captive forest musk deer populations and its implication for conservation‐sciencedirect. Biochemical Systematics & Ecology, 37(3), 166–173. 10.1016/j.bse.2009.04.001

[eva13532-bib-0010] Guo, S. W. , & Thompson, E. A. (1992). Performing the exact test of hardy‐Weinberg proportion for multiple alleles. Biometrics, 48(2), 361–372. 10.2307/2532296 1637966

[eva13532-bib-0011] Hu, Y. B. , Zhan, X. J. , Qi, D. W. , & Wei, F. (2010). Spatial genetic structure and dispersal of giant pandas on a mountain‐range scale. Conservation Genetics, 11, 2145–2155. 10.1007/s10592-010-0100-1

[eva13532-bib-0012] Huang, J. (2015). The development and the applications of universal marker system based on microsatellites of giant panda (Ailuropoda melanoleuca). Sichuan University.

[eva13532-bib-0013] Hull, V. , Roloff, G. , Zhang, J. D. , Liu, W. , Zhou, S. Q. , Huang, J. Y. , Xu, W. H. , Ouyang, Z. Y. , Zhang, H. M. , & Liu, J. G. (2014). A synthesis of giant panda habitat selection. Ursus, 25(2), 148–162. 10.2192/URSUS-D-13-00011.1

[eva13532-bib-0014] Ji, J. F. (2014). Microsatellite analysis about genetic diversity of Giant panda in Liangshan and Qinling Mountains. Guangxi Normal University. 10.7666/d.Y2585076

[eva13532-bib-0015] Ji, Y. , & Jiang, Z. (2011). Genetic diversity, population genetic structure and demographic history of przewalski's gazelle (*Procapra przewalskii*): Implications for conservation. Conservation Genetics, 12(6), 1457–1468. 10.1007/s10592-011-0244-7

[eva13532-bib-0016] Kvist, L. , Giralt, D. , Valera, F. , Hoi, H. , & Lovaszi, P. (2015). Population decline is accompanied by loss of genetic diversity in the lesser Grey shrike *lanius minor* . Ibis, 153(1), 98–109. 10.1111/j.1474-919X.2010.01091.x

[eva13532-bib-0017] Li, M. , Wei, F. W. , Rao, G. , Fang, S. G. , & Feng, Z. J. (2001). Application of noninvasive sampling in conservation genetics. Current Zoology, 47(3), 338–342. 10.3969/j.issn.1674-5507.2001.03.016

[eva13532-bib-0018] Li, Y. Z. , Xu, X. , Shen, F. J. , Zhang, W. P. , Zhang, Z. H. , Hou, R. , & Yue, B. S. (2010). Development of new tetranucleotide microsatellite loci and assessment of genetic variation of giant panda in two largest giant panda captive breeding populations. Journal of Zoology, 282(1), 39–46. 10.1111/j.1469-7998.2010.00707.x

[eva13532-bib-0019] Librado, P. , & Rozas, J. (2009). Dnasp v5: A software for comprehensive analysis of DNA polymorphism data. Bioinformatics, 25(11), 1451–1452. 10.1093/bioinformatics/btp187 19346325

[eva13532-bib-0020] Loucks, C. J. , Lu, Z. , Dinerstein, E. , Wang, H. , Olson, D. M. , Zhu, C. , & Wang, D. (2001). Giant pandas in a changing landscape. Science, 294(5546), 1465. 10.1126/science.1064710 11711657

[eva13532-bib-0021] Lu, Z. , Johnson, W. E. , Menotti‐Raymond, M. , Yuhki, N. , Martenson, J. S. , Mainka, S. , Huang, S. Q. , Zheng, Z. H. , Li, G. H. , Pan, W. S. , Mao, X. R. , & O'Brien, S. J. (2001). Patterns of genetic diversity in remaining giant panda populations. Conservation Biology, 15(6), 1596–1607. 10.1046/j.1523-1739.2001.00086.x

[eva13532-bib-0022] Marshall, T. C. , Slate, J. , Kruuk, L. , & Pemberton, J. M. (2010). Statistical confidence for likelihood‐based paternity inference in natural populations. Molecular Ecology, 7(5), 639–655. 10.1046/j.1365-294x.1998.00374.x 9633105

[eva13532-bib-0023] National Park Administration . (2019). National Forestry and grassland administration (National Park Administration). Master plan for Giant Panda National Park.

[eva13532-bib-0024] O'Brien, S. J. , Pan, W. S. , & Lu, Z. (1994). Pandas, people and policy. Nature, 369(19), 179–180. 10.1038/369179a0 8183334

[eva13532-bib-0025] Park, S. D. E. (2001). Trypanotolerance in west African cattle and the population genetic effects of selection. University of Dublin.

[eva13532-bib-0026] Peakall, R. , & Smouse, P. E. (2012). GenAlEx 6.5: Genetic analysis in excel. Population genetic software for teaching and research an update. Bioinformatics, 28(19), 2537–2539. 10.1093/bioinformatics/bts460 22820204 PMC3463245

[eva13532-bib-0027] Piggott, M. P. , Banks, S. C. , Stone, N. , Banffy, C. , & Taylor, A. C. (2006). Estimating population size of endangered brush‐tailed rock‐wallaby (*Petrogale penicillata*) colonies using faecal DNA. Molecular Ecology, 15(1), 81–91. 10.1111/j.1365-294X.2005.02783.x 16367832

[eva13532-bib-0028] Price, M. (2015). Mastering ArcGIS. WCB/McGraw‐Hill.

[eva13532-bib-0029] Pritchard, J. K. , Stephens, M. J. , & Donnelly, P. J. (2000). Inference of population structure using multilocus genotype data. Genetics, 155(2), 945–959. 10835412 10.1093/genetics/155.2.945 PMC1461096

[eva13532-bib-0030] Qin, S. S. , Dang, N. X. , Nie, H. , & Cao, Y. (2017). Evolutionary analysis of mitochondrial genomes of giant pandas from different genetic group. Genomics and Applied Biology, 36(9), 3696–3703.

[eva13532-bib-0031] Qing, J. (2016). Study on the habitat fragmentation and designing corridors of giant panda in Sichuan province . China West Normal University.

[eva13532-bib-0032] Raymond, M. , & Rousset, F. (1995). Genepop (version 1.2): Population genetics software for exact tests and ecumenicism. Journal of Heredity, 86(3), 248–249. 10.1046/j.1420-9101.1995.8030385.x

[eva13532-bib-0033] Selkoe, K. A. , & Toonen, R. J. (2010). Microsatellites for ecologists: A practical guide to using and evaluating microsatellite markers. Ecology Letters, 9(5), 615–629. 10.1111/j.1461-0248.2006.00889.x 16643306

[eva13532-bib-0034] Shan, L. , Hu, Y. B. , Zhu, L. F. , Yan, L. , Wang, C. D. , Li, D. S. , Jin, X. L. , Zhang, C. L. , & Wei, F. W. (2014). Large‐scale genetic survey provides insights into the captive management and reintroduction of giant pandas. Molecular Biology and Evolution, 31(10), 2663–2671. 10.1093/molbev/msu210 25015646

[eva13532-bib-0035] Shen, F. J. , Zhang, Z. H. , He, W. , Yue, B. S. , Zhang, A. J. , Zhang, L. , Hou, R. , Wang, C. D. , & Watanabe, T. (2010). Microsatellite variability reveals the necessity for genetic input from wild giant pandas (*Ailuropoda melanoleuca*) into the captive population. Molecular Ecology, 18(6), 1061–1070. 10.1111/j.1365-294X.2009.04086.x 19222753

[eva13532-bib-0036] Shi, X. W. , Zhang, J. D. , & Ouyang, Z. Y. (2016). Research progress on population investigation methods for wild giant panda. Acta Ecologica Sinica, 36(23), 7528–7537. 10.5846/stxb201510292185

[eva13532-bib-0037] Sichuan Provincial Forestry Department . (2015). The pandas of Sichuan: The fourth survey report on Giant panda in Sichuan Province. Sichuan Science and Technology Press.

[eva13532-bib-0038] State Forestry Administration . (2006). The Third National Survey Report on Giant panda in China. Science Press.

[eva13532-bib-0039] Tamura, K. , Peterson, D. , Peterson, N. , Stecher, G. , Nei, M. , & Kumar, S. (2011). MEGA5: Molecular evolutionary genetics analysis using maximum likelihood, evolutionary distance, and maximum parsimony methods. Molecular Biology and Evolution, 28(10), 2731–2739. 10.1093/molbev/msr121 21546353 PMC3203626

[eva13532-bib-0040] Valière, N. (2010). GIMLET: A computer program for analyzing genetic individual identification data. Molecular Ecology Notes, 2(3), 377–379. 10.1046/j.1471-8286.2002.00228.x

[eva13532-bib-0041] Vanhala, T. , Tuiskula‐Haavisto, M. , Elo, K. , Vilkki, J. , & Mki‐Tanila, A. (1998). Evaluation of genetic variability and genetic distances between eight chicken lines using microsatellite markers. Poultry Science, 77(6), 783–790. 10.1093/ps/77.6.783 9628523

[eva13532-bib-0042] Van‐Oosterhout, C. , Hutchinson, W. F. , Wills, D. P. M. , & Shipley, P. (2004). Micro‐checker software for identifying and correcting genotyping errors in microsatellite data. Molecular Ecology Notes, 4(3), 535–538. 10.1111/j.1471-8286.2004.00684.x

[eva13532-bib-0043] Waits, L. P. , Luikart, G. , & Taberlet, P. (2001). Estimating the probability of identity among genotypes in natural populations: Cautions and guidelines. Molecular Ecology, 10, 249–256. 10.1046/j.1365-294X.2001.01185.x 11251803

[eva13532-bib-0044] Wang, D. , Hu, Y. B. , Ma, T. X. , Nie, Y. G. , Xie, Y. , & Wei, F. W. (2016). Noninvasive genetics provides insights into the population size and genetic diversity of an Amur tiger population in China. Integrative Zoology, 11(1), 16–24. 10.1111/1749-4877.12176 26663614

[eva13532-bib-0045] Wang, E. Z. (2019). Silver carp genetic diversity from four famous domestic fishes origin field revealed by mitochondrial DNA D‐loop sequence . South‐Central University for Nationalities.

[eva13532-bib-0046] Wei, F. W. , Hu, Y. B. , Zhu, L. F. , Bruford, M. W. , Zhan, X. J. , & Zhang, L. (2012). Black and white and read all over: The past, present and future of giant panda genetics. Molecular Ecology, 21, 5660–5674. 10.1111/mec.12096 23130639

[eva13532-bib-0047] Wei, W. , Swaisgood, R. R. , Pilfold, N. W. , Owen, M. A. , Dai, Q. , Wei, F. , Han, H. , Yang, Z. , Yang, X. , Gu, X. D. , Zhang, J. D. , Yuan, S. B. , Hong, M. S. , Tang, J. F. , Zhou, H. , He, K. , & Zhang, Z. J. (2020). Assessing the effectiveness of China's panda protection system. Current Biology, 30(7), 1280–1286. 10.1016/j.cub.2020.01.062 32197077

[eva13532-bib-0048] Wright, S. (1990). Evolution in mendelian populations. Bulletin of Mathematical Biology, 52(2), 241–295. 10.1007/BF02459575 2185860

[eva13532-bib-0049] Xu, W. H. , Pimm, S. L. , Du, A. , Su, Y. , Fan, X. Y. , An, L. , Liu, J. G. , & Ouyang, Z. Y. (2019). Transforming protected area management in China. Trends in Ecology & Evolution, 34(9), 762–766. 10.1016/j.tree.2019.05.009 31301875

[eva13532-bib-0050] Xu, W. H. , Xiao, Y. , Zhang, J. J. , Yang, W. , Zhang, L. , Hull, V. , Wang, Z. , Zheng, H. , Liu, J. G. , Polasky, S. , Jiang, L. , Xiao, Y. , Shi, X. W. , Rao, E. , Lu, F. , Wang, X. K. , Daily, G. C. , & Ouyang, Z. Y. (2017). Strengthening protected areas for biodiversity and ecosystem services in China. Proceedings of the National Academy of Sciences of the United States of America, 114(7), 1601–1606. 10.1073/pnas.1620503114 28137858 PMC5321011

[eva13532-bib-0051] Ya, H. E. , Wang, Z. H. , & Wang, X. M. (2014). Genetic diversity and population structure of a Sichuan sika deer (*Cervus sichuanicus*) population in Tiebu nature reserve based on microsatellite variation. Zoological Research, 35(6), 528–536. 25465089 10.13918/j.issn.2095-8137.2014.6.528 PMC4790281

[eva13532-bib-0052] Yang, B. (2013). The giant panda (Ailuropoda melanoleuca) mitochondrial genome research and genetic diversity analysis of the captive population. Sichuan Agricultural University.

[eva13532-bib-0053] Yang, Z. S. , Gu, X. D. , Nie, Y. G. , Huang, F. , Huang, Y. , Dai, Q. , Hu, Y. B. , Yang, Y. , Zhou, X. , Zhang, H. M. , Yang, X. Y. , & Wei, F. W. (2018). Reintroduction of the giant panda into the wild: A good start suggests a bright future. Biological Conservation, 217, 181–186. 10.1016/j.biocon.2017.08.012

[eva13532-bib-0054] Yeh, F. C. , Yang, R. , Boyle, T. J. , Ye, Z. , & Xiyan, J. M. (2000). Popgene32, Microsoft windows based freeware for population genetic analysis, version 1.32. Molecular Biology and Biotechnology Centre, University of Alberta.

[eva13532-bib-0056] Zhan, X. J. , Li, M. , Zhang, Z. J. , Goosens, B. , Chen, Y. P. , Wang, H. J. , Bruford, M. W. , & Wei, F. W. (2006). Molecular censusing doubles giant panda population estimate in a key nature reserve. Current Biology, 16, 451–452. 10.1016/j.cub.2006.05.042 16781997

[eva13532-bib-0057] Zhang, B. W. , Li, M. , Zhang, Z. J. , Goossens, B. , Zhu, L. F. , Zhang, S. N. , Hu, J. C. , Bruford, M. W. , & Wei, F. W. (2007). Genetic viability and population history of the giant panda, putting an end to the “evolutionary dead end”? Molecular Biology and Evolution, 24(8), 1801–1810. 10.1093/molbev/msm099 17513881

[eva13532-bib-0058] Zhang, Y. K. , Wu, Y. J. , Zhang, Q. Y. , Ran, J. H. , & Price, M. L. (2018). Distribution of a giant panda population influenced by land cover. The Journal of Wildlife Management, 82(6), 1199–1209. 10.1002/jwmg.21477

[eva13532-bib-0059] Zhao, C. , Yue, B. S. , Ran, J. H. , Moermond, T. , Hou, N. , Yang, X. Y. , & Gu, X. D. (2017). Relationship between human disturbance and endangered giant panda *Ailuropoda melanoleuca* habitat use in the Daxiangling Mountains. Oryx, 51, 146–152. 10.1017/S0030605315000800

[eva13532-bib-0060] Zhou, Y. Y. , Hai‐Rui, D. , Xue, Y. D. , Di‐Qiang, L. I. , Feng, J. C. , & Zhang, Y. G. (2015). Genetic diversity analysis of microsatellite DNA in snow leopard (*Panthera uncia*). Chinese Journal of Zoology, 50(2), 161–168.

[eva13532-bib-0061] Zou, F. D. , Yue, B. S. , Xu, L. , & Zhang, Y. Z. (2005). Isolation and characterization of microsatellite loci from forest musk deer (*Moschus berezovskii*). Zoological Science, 22(5), 593–598. 10.1021/ja0562447 15930833

