# Supplementary material for: Large‐scale genetic surveys for main extant population of wild giant panda (Ailuropoda melanoleuca) reveals an urgent need of human management
Source: Evol Appl. 2023 Feb 5;16(3):738–49. doi: 10.1111/eva.13532 (PMC10033846; doi:10.1111/eva.13532)
Supplement: Supplementary file 1 — Figures S1‐S3 [file EVA-16-738-s001.docx]

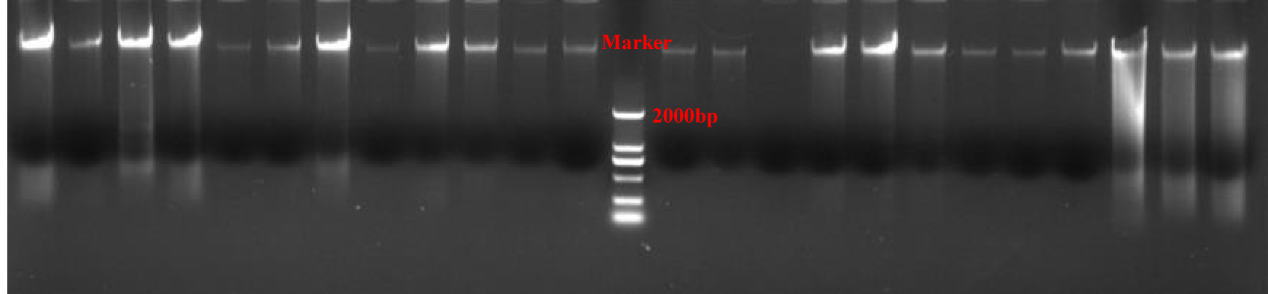


**Figure S1 DNA electrophoresis results of giant panda some feces samples**


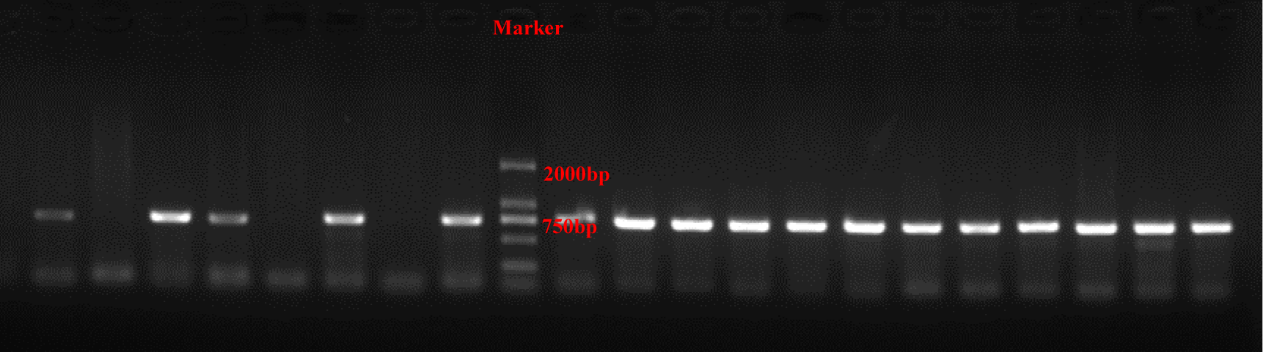


**Figure S2 Electrophoretic detection of PCR amplification products of mitochondrial control region**


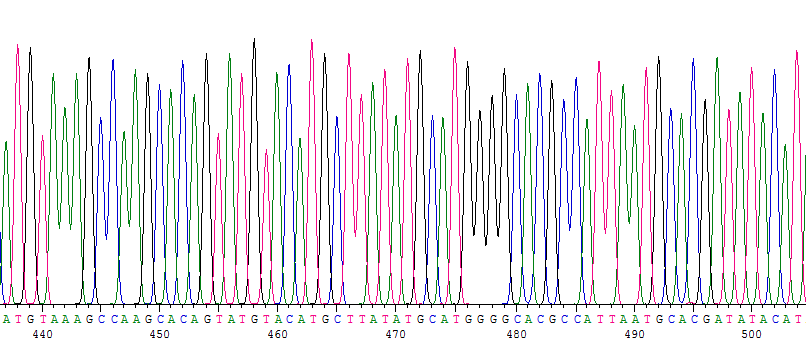


a


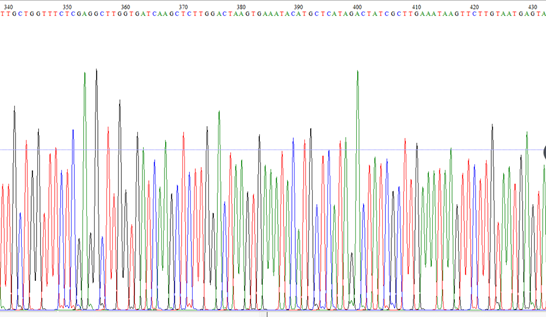


b


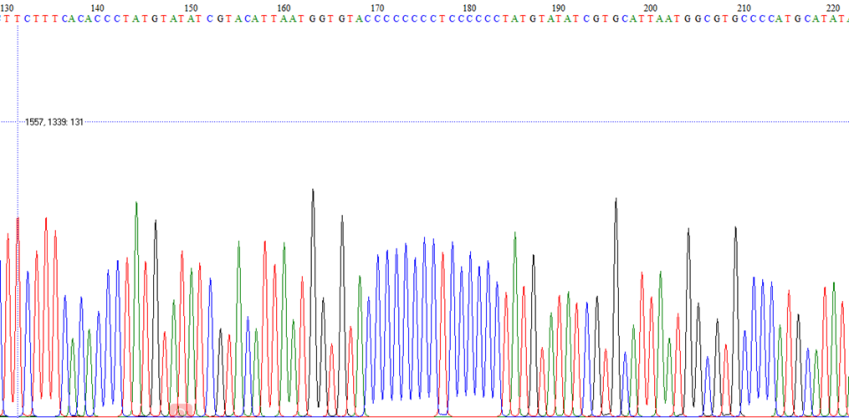


c

**Figure S3 Sequencing map of mitochondrial control region of giant pandas (a:MB、b:MG、c:HZG)**
